# The Galaxy platform for accessible, reproducible and collaborative biomedical analyses: 2022 update

**DOI:** 10.1093/nar/gkac247

**Published:** 2022-04-21

**Authors:** The Galaxy Community, Enis Afgan, Anton Nekrutenko, Bjórn A Grüning, Daniel Blankenberg, Jeremy Goecks, Michael C Schatz, Alexander E Ostrovsky, Alexandru Mahmoud, Andrew J Lonie, Anna Syme, Anne Fouilloux, Anthony Bretaudeau, Anton Nekrutenko, Anup Kumar, Arthur C Eschenlauer, Assunta D DeSanto, Aysam Guerler, Beatriz Serrano-Solano, Bérénice Batut, Björn A Grüning, Bradley W Langhorst, Bridget Carr, Bryan A Raubenolt, Cameron J Hyde, Catherine J Bromhead, Christopher B Barnett, Coline Royaux, Cristóbal Gallardo, Daniel Blankenberg, Daniel J Fornika, Dannon Baker, Dave Bouvier, Dave Clements, David A de Lima Morais, David Lopez Tabernero, Delphine Lariviere, Engy Nasr, Enis Afgan, Federico Zambelli, Florian Heyl, Fotis Psomopoulos, Frederik Coppens, Gareth R Price, Gianmauro Cuccuru, Gildas Le Corguillé, Greg Von Kuster, Gulsum Gudukbay Akbulut, Helena Rasche, Hans-Rudolf Hotz, Ignacio Eguinoa, Igor Makunin, Isuru J Ranawaka, James P Taylor, Jayadev Joshi, Jennifer Hillman-Jackson, Jeremy Goecks, John M Chilton, Kaivan Kamali, Keith Suderman, Krzysztof Poterlowicz, Le Bras Yvan, Lucille Lopez-Delisle, Luke Sargent, Madeline E Bassetti, Marco Antonio Tangaro, Marius van den Beek, Martin Čech, Matthias Bernt, Matthias Fahrner, Mehmet Tekman, Melanie C Föll, Michael C Schatz, Michael R Crusoe, Miguel Roncoroni, Natalie Kucher, Nate Coraor, Nicholas Stoler, Nick Rhodes, Nicola Soranzo, Niko Pinter, Nuwan A Goonasekera, Pablo A Moreno, Pavankumar Videm, Petera Melanie, Pietro Mandreoli, Pratik D Jagtap, Qiang Gu, Ralf J M Weber, Ross Lazarus, Ruben H P Vorderman, Saskia Hiltemann, Sergey Golitsynskiy, Shilpa Garg, Simon A Bray, Simon L Gladman, Simone Leo, Subina P Mehta, Timothy J Griffin, Vahid Jalili, Vandenbrouck Yves, Victor Wen, Vijay K Nagampalli, Wendi A Bacon, Willem de Koning, Wolfgang Maier, Peter J Briggs

## Abstract

Galaxy is a mature, browser accessible workbench for scientific computing. It enables scientists to share, analyze and visualize their own data, with minimal technical impediments. A thriving global community continues to use, maintain and contribute to the project, with support from multiple national infrastructure providers that enable freely accessible analysis and training services. The Galaxy Training Network supports free, self-directed, virtual training with >230 integrated tutorials. Project engagement metrics have continued to grow over the last 2 years, including source code contributions, publications, software packages wrapped as tools, registered users and their daily analysis jobs, and new independent specialized servers. Key Galaxy technical developments include an improved user interface for launching large-scale analyses with many files, interactive tools for exploratory data analysis, and a complete suite of machine learning tools. Important scientific developments enabled by Galaxy include Vertebrate Genome Project (VGP) assembly workflows and global SARS-CoV-2 collaborations.

## Graphical Abstract

**Graphical Abstract:**
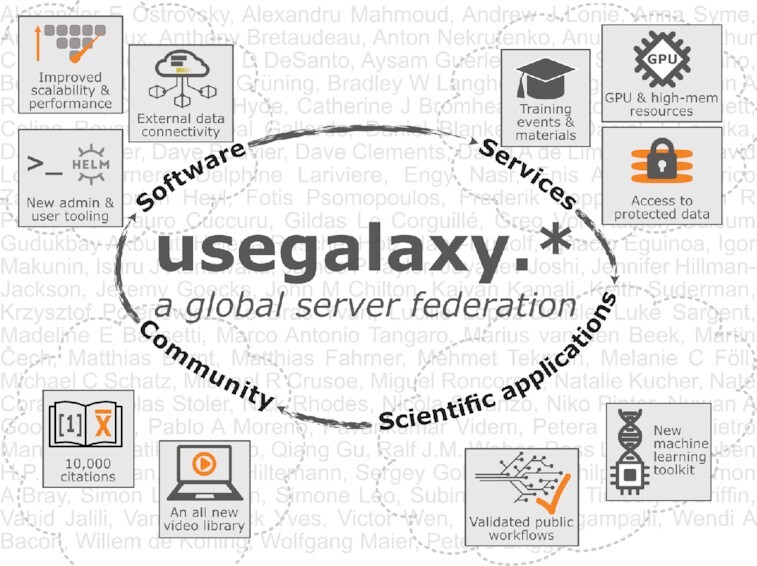
The Galaxy Project is represented by a combination of software, managed services, application of the software for solving scientific analyses, and vibrant world-wide community. In turn, each area is characterized by new depicted capabilities.

## INTRODUCTION

Rapid growth in FAIR (1) data, and an expanding range of open source analysis software packages, offer rich research opportunities in data intensive fields such as genome science. Galaxy offers powerful and practical solutions for analyzing this data by providing access to extensive hardware, tools, and data that can be adopted with relatively minimal training. The open source software allows scientists to efficiently manage their own data, and to share transparent, reproducible analyses. More than 8000 popular analysis software packages have been integrated with Galaxy and their use is supported via numerous topic-based training resources. The growing breadth of tools available in Galaxy enables diverse types of analysis and because all data manipulations are performed via tools (as opposed to ad-hoc scripts or manual editing), reproducibility is ensured. Galaxy also offers an interactive workflow manager (2) that makes efficient use of compute infrastructure, and comes preloaded with access to terabytes of reference data. There is also an extensible visualization framework (https://usegalaxy.org/visualizations) with built-in track-based genome visualizations (https://galaxyproject.org/learn/visualization/), multiple types of charts (barcharts, scatterplots, line charts, etc; https://galaxyproject.org/learn/visualization/charts/), as well as more specialized visualization tools such as Cytoscape (3), NGL molecular visualizations (4), and geographic maps from OpenLayers.

In addition to software, the project community offers access to free computing services on large research infrastructure around the world with most prominent installations residing in Australia, Europe, and the United States. Self-hosted cloud deployments are also supported via the NHGRI AnVIL infrastructure (https://anvilproject.org/) (5). All the software and services are accompanied with a growing library of training materials and events (6).

**Figure 1.**
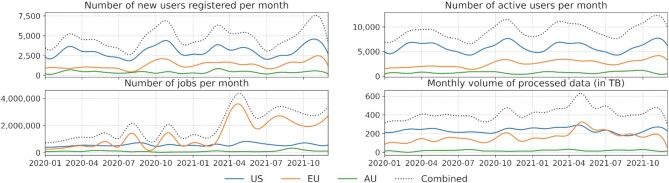
Usage of the usegalaxy servers in Australia (AU), Europe Union (EU) and the United States (US). Large compute infrastructure is available to anyone, for free, without any configuration and it spans the world (more below). User acquisition, user retention, and user activity are captured. A dip in usage captured at the right hand side of some diagrams is cyclical, due to the end of the calendar year. A significant increase in the number of monthly jobs in the EU is due to the start of analyzing SARS-CoV-2 data (more below).

**Figure 2.**
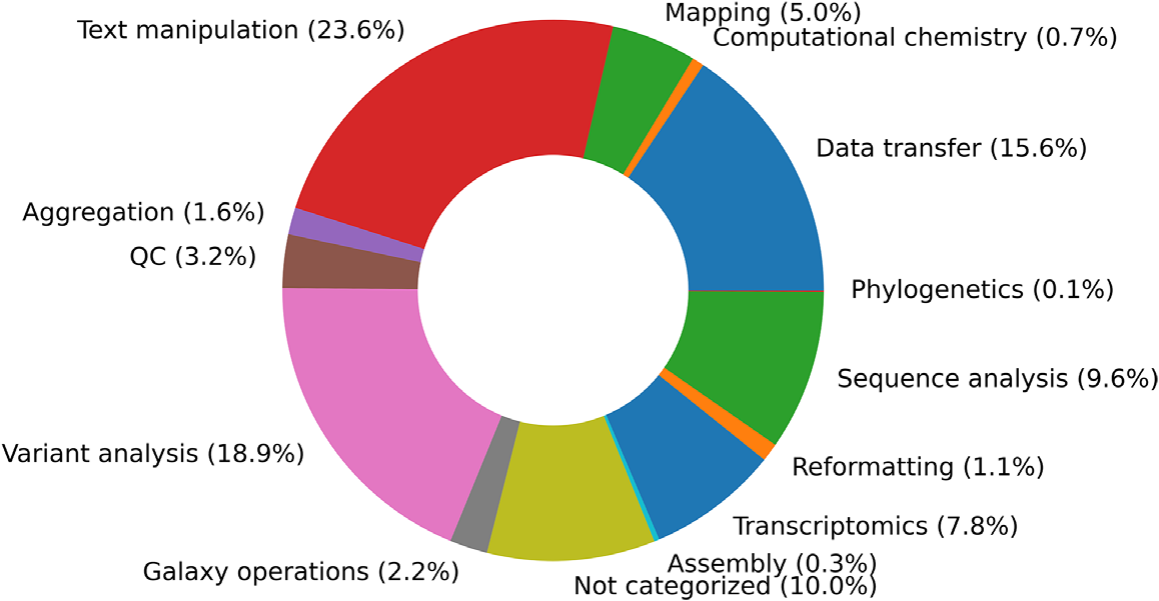
Categorization of the type of tools executed by users across the three most popular usegalaxy servers.

Started in 2005 (7), the Galaxy project is now sustained by a strong, global community of users, educators, developers and administrators. The size of this community is continuing to grow across all areas of the Galaxy ecosystem. Highlighted in Figure 1 is the usage of the free services by the researchers while Figure 2 summarizes the categories of tools researchers use via Galaxy most frequently. We believe the size and diversity of this community is what sets Galaxy apart from alternative data analysis platforms. For a detailed perspective on the alternative workflow management systems and a comparison of technical features, please see Wratten *et al.* (8).

In the remainder of this paper, we provide details on some of the most relevant updates to show how the Galaxy project is growing and adapting to the changing and complicated landscape of computing in open science. As will be seen, the work reported here is only possible because multiple independent research groups and a global community of contributors collaborate efficiently to support the project (https://galaxyproject.org/).

## NEW SOFTWARE FEATURES AND ENHANCEMENTS

Genomic data analyses are continuing to push the boundaries in terms of the size of the data generated and number of samples processed. This is coupled with an increased adoption of cloud computing services for hosting the data and more stringent restrictions on data movement. The Galaxy project has developed a number of new features to accommodate these trends.

### Ability to browse external data repositories

Public and private data repositories are a growing trend for hosting, aggregating, and sharing data. Galaxy can now represent these remote data resources as a UI-browsable filesystem, which users can upload to and download from. Remote file source plugins bundled with Galaxy include AWS S3 storage service, Google Cloud Storage, Google Drive, AnVIL, and Dropbox. To add a new data source module to Galaxy, one need only find or write a client library for the desired source that implements the PyFilesystem2 interface, and configure Galaxy to provision it with appropriate user credentials.

### Initial support for batch operations

Dataset collections allow the same operations, such as executing a tool or workflow, to be performed on a set of datasets. Collections have been enhanced to allow converting the datatype for all items in a collection as a batch operation. Another major development is in the rule builder, which allows data being imported into a collection to be structured based on defined rules (e.g. create a collection of dataset pairs from a list of accessions). The rule builder now also retains memory of the most recent rules that it ran, allowing users to re-run this saved rules list on a new collection of datasets.

### Modernizing the framework

Since its inception, Galaxy used the Web Server Gateway Interface (WSGI) convention for its web server functionality through various low-level Python libraries. Modern best practices, however, recommend the use of features such as standard asynchronous computing framework (asyncio), type annotations (mypy), data validation (pydantic) and live documentation (OpenAPI) for increased efficiency and maintainability. To utilize these advances, Galaxy has adopted the FastAPI web framework and can now be served as an Asynchronous Server Gateway Interface (ASGI) application. This transition has resulted in a simplified development experience with auto-generated documentation, more robust parsing of parameters, better error handling and the ability to handle more user requests with fewer server resources. Importantly, the API schema is automatically up-to-date and correct, and can be used to generate client libraries in many different programming languages. Finally, these updates provide the groundwork for interactive applications and notifications using the websocket protocol.

An additional framework upgrade is the adoption of a message queue for long-running tasks. As the number of datasets that Galaxy must support continues to grow, it has become untenable to perform even trivial processing of such datasets in the web request-response cycle itself. We have extended Galaxy with support for Celery tasks, which allows such long-running operations to be offloaded from the server request. Slow operations, such as deleting datasets and exporting histories, have been transitioned to this background processing model with more planned. These and other optimizations have substantially increased job throughput and provided a thousand-fold client speedup when handling dataset collections with 100 000 elements.

### Workflow best practices, invocations, and reports

**Figure 3.**
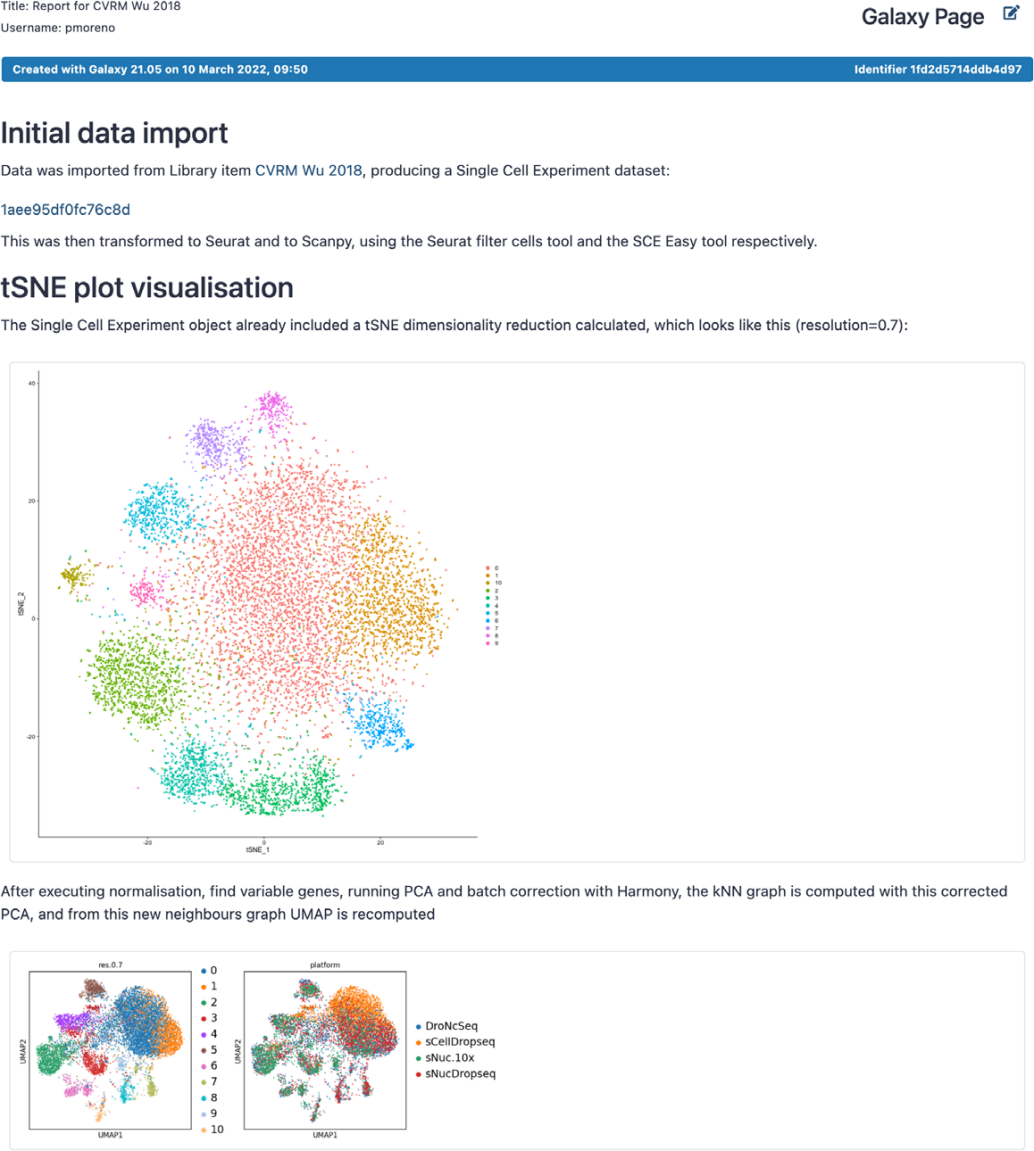
A sample workflow report, showing tSNE and UMAP plots of single cell expression data, automatically generated and formatted based on the outputs of a workflow.

Galaxy’s workflow editor offers powerful capabilities to formulate multi-step pipelines for analyzing thousands of samples, all from a graphical web interface. The editor has been upgraded to offer researchers automated advice for adhering to workflow development best practices. The advice includes support for automatically upgrading legacy workflow inputs to the current format, warnings about disconnected inputs, missing metadata for inputs, missing outputs, and missing license and creator metadata. Additionally, a new workflow invocation component was added that displays historic and currently executing workflow runs, while displaying input parameters, input datasets, input dataset collections, outputs, all workflow steps, jobs and subworkflows. The invocations and any changes in the history are reflected in the workflow component and vice-versa, bringing a powerful way to manage the complexity of large workflows that output many thousands of datasets. Finally, the workflow experience has been enhanced with workflow reports. Based on a reusable template, workflow reports can be used to summarize a workflow run in a structured document and downloaded as a PDF report (Figure 3).

### A complete administrator’s toolset

The growth of the Galaxy user community has also driven a professionalization of the Galaxy Administrator, and we teach ever larger courses to Galaxy Admins in response. This has likewise resulted in the development of numerous utilities and projects to make their lives easier. For example, gxadmin (https://galaxyproject.github.io/gxadmin) is a collection of commonly used SQL queries that provide infrastructure statistics for reporting and debugging. Nebulizer (https://github.com/pjbriggs/nebulizer) and Ephemeris (https://github.com/galaxyproject/ephemeris) help administrators automate tasks like importing data, installing tools, and managing users. parsec was created to expose all of the Galaxy API as a set of command line tools that can be composed in a UNIX-like manner.

### Galaxy Helm chart

We have developed a new Kubernetes Helm chart for Galaxy that abstracts the complex mechanics of deploying Galaxy into a single, highly-configurable package. The chart uses a recommended and reproducible set of technologies for deployment and management of Galaxy in development, testing or large-scale production scenarios. The chart supports versioned configuration, zero downtime upgrades, application scaling, and comes pre-configured with several hundred vetted tools, reference genomes, and monitoring dashboards.

## MANAGED SERVICES FOR THE WORLD

A characteristic of Galaxy is that it comes with ‘batteries included’, meaning that in addition to the software and training materials, it supports access to powerful and instantly accessible services allowing anyone to use the software without setup or a fee. Here, we describe two new major features that continue to advance the availability of these services for training events and analysis of protected data.

### Towards unification of public Galaxy servers

Usegalaxy.* is a federation of free, public Galaxy servers that adhere to a set of common standards. There are currently several such servers (https://galaxyproject.org/use/), all of which are provided and maintained by community members. Three most popular reside in Australia (AU), the European Union (EU), and the United States (US), and run on national compute infrastructures with recent advances including support for GPUs and high-memory machines. These resource advances are paving a path for novel or larger analyses using these servers as opposed to requiring groups to install and maintain their own. Another key new feature of the select usegalaxy.* servers is support for Training Infrastructure as a Service (TIaaS) (9). With TIaaS, training instructors can reserve dedicated infrastructure for a workshop through a web request form, with participants having their jobs prioritized for execution on that infrastructure. Upon completion of the event, users retain their data and analysis histories on the given server and can revisit it later. This ensures that training events are unperturbed by unpredictable server load from other users. To date, TIaaS has provided priority queue access for >285 events around the world, helping over 12 000 students learn bioinformatics and science on the Galaxy platform. Nearly 130 000 compute hours were provided to support these training events.

### Galaxy service for protected and private data

One of the biggest changes in the Galaxy ecosystem is general availability of a Galaxy service for use with protected and private datasets. In the context of the NHGRI AnVIL project (5), we have implemented new capabilities that enable anyone to securely access Galaxy alongside patient records and >300 000 genomes, eliminating the need to download, store, and protect that data locally. AnVIL operates with US FedRAMP certification (10). These strengthened privacy guarantees also open a door for researchers to upload their private data to this resource. This is particularly appealing for smaller institutions that do not have the resources to build their own secure data center, and can instead launch their own instances of Galaxy within a highly scalable cloud-computing environment, broadening and democratizing accessibility of data analysis options. This solution also replaces earlier implementations of Galaxy-on-the-cloud (11).

## SCIENTIFIC APPLICATIONS AND USES

We are strong believers in the ‘Eat your own dog food’ expression as a method of ensuring that the software and services built by the project truly solve real-world analysis needs. Here we highlight a few of the ongoing scientific efforts that use Galaxy and demonstrate how the above described advances facilitate adoption of Galaxy for analysis needs.

### A community-curated repository of high-quality workflows

The Intergalactic Workflow Commision (IWC; https://github.com/galaxyproject/iwc) is a new community group that develops, collects, and improves Galaxy workflows. The workflows are maintained in an open repository as reusable components, and include such diverse topics as variant analysis of SARS-CoV-2 data and free energy calculations for molecular dynamics simulations. Anyone can contribute a workflow, which is then peer reviewed by the IWC according to pre-set guidelines. Once accepted, each workflow is continuously checked for best-practice conformance and tested during each new Galaxy release. The workflows are also automatically published to Dockstore (12) and WorkflowHub (13) and optionally synchronized to a list of Galaxy servers.

### Analysis of public SARS-CoV-2 data

After nearly two years of the global COVID-19 pandemic and numerous virus strains, pathogen genomic surveillance has become an essential public service. The current knowledge about the evolutionary dynamics of SARS-CoV-2 comes primarily from genome assemblies (14). However, the availability of the read-level datasets used to build these assemblies lags behind the number of complete genome assemblies, making it impossible to confirm or investigate these genomes further, such as to further refine sequencing errors or detect potential co-infections. In addition, only a fraction of available read-level datasets are useful for transmission analysis because they lack necessary metadata (https://galaxyproject.org/projects/covid19/samples/). The Galaxy project now continuously ingests and analyzes high quality read-level datasets on public infrastructure, providing a platform for global pathogen monitoring (15). The workflows and data used for this monitoring is available at https://galaxyproject.org/projects/covid19/.

### A machine learning toolkit

**Figure 4.**
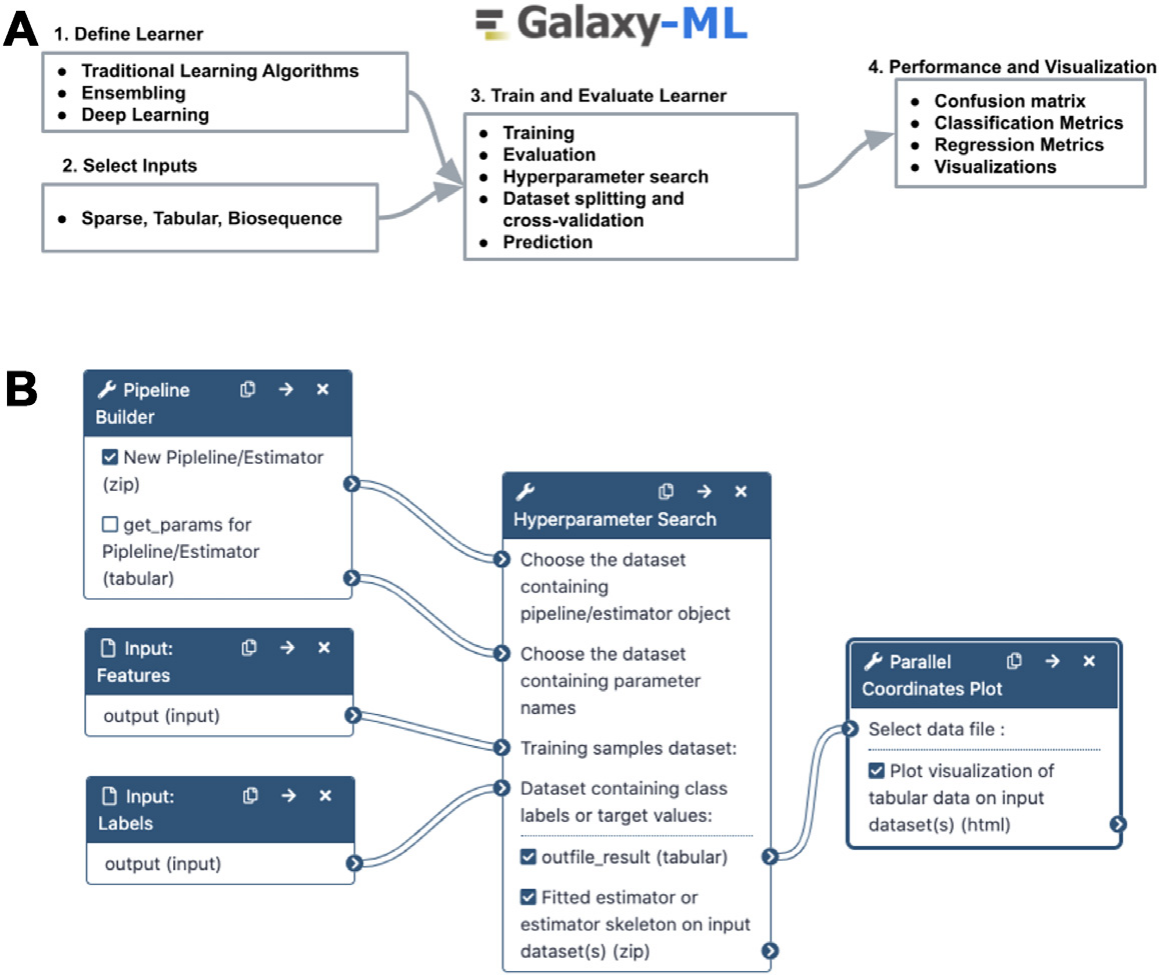
(**A**) The Galaxy-ML toolkit provides all the tools necessary to define a learner, train it, evaluate it, and visualize its performance. (**B**) A Galaxy workflow to create a learner using a pipeline, perform hyperparameter search and visualize the results.

Galaxy-ML (16) (Figure 4) is a new toolkit for Galaxy that features a large and general-purpose suite of supervised machine learning tools. With Galaxy’s web-based user interface, an entire machine learning pipeline from normalization, feature selection, model definition, hyperparameter optimization and cross-fold evaluation can be created and applied to large datasets using only a web browser. By leveraging analysis tools already available in Galaxy, comprehensive end-to-end analyses can be performed, beginning with primary analysis of -omics, imaging, or other large biomedical datasets and continuing to downstream machine learning tools that build and evaluate predictive machine learning models from features extracted from the primary data.

## A VIBRANT GLOBAL COMMUNITY

A core strength of the Galaxy project is its bottom-up structure supported by a worldwide community of contributors. The following paragraphs outline some of their accomplishments.

### JXTX: James P. Taylor foundation for open science

James Taylor, the original developer and co-founder of the Galaxy project, died unexpectedly on 2 April 2020 (17). While we may never fully recover from this shock, the project established JXTX: The James P. Taylor foundation for open science. The foundation provides support for graduate students to attend conferences in computational biology and data science to present their work and form connections with other researchers. To date, the foundation provided support to 20 students to present their work at the Cold Spring Harbor Conferences on Biological Data Science (2020) and Genome Informatics (2021). Please help us to continue provide support by donating at https://jxtxfoundation.org/.

### 10,000 publications

In 2020, the number of publications that cite the Galaxy project surpassed 10 000. The Galaxy Publication Library https://galaxyproject.org/publication-library/ tracks publications that use, extend, implement, or reference Galaxy or Galaxy-based platforms and represents a view into the global Galaxy community. Notably, the majority of publications (59%) reference Galaxy in their methods, implying Galaxy is being used as a common tool in reaching experiment results. Researchers are increasingly using managed Galaxy services (20%, up from 15% in 2015 with local servers dropping from 12% in 2015 to 9% in 2020). Researchers have also increasingly spread their use across a growing number of public Galaxy servers: in 2020, 20% are using public servers and 10% are using usegalaxy.org, compared to 15% using each type of service in 2015. Finally, publishing in open access journals has been on the rise since 2017 (74% of publications in 2017 versus 86% in 2020) with the most popular choices in 2020 being PLOS ONE, Scientific reports, NAR, BMC Genomics and Bioinformatics.

### Galaxy Training Network

Much of Galaxy is backed by the Galaxy Training Network (GTN), a strong and vibrant community centered around a central repository (https://training.galaxyproject.org/) of training material spanning multiple scientific domains (6). With over 220 community members contributing content, the GTN training repository contains over 230 tutorials covering 16 scientific topics, and 6 technical topics (e.g. developer, administrator, and teacher training). In addition, the GTN organizes training events. With the ongoing SARS-CoV-2 pandemic, training events have increasingly occurred in an asynchronous, virtual setting via pre-recorded training videos that participants can work through at their own pace, with support from instructors available online. An example event was the GTN Smörgåsbord in March 2021, a 5-day 24/7 training event involving 60 instructors and 1,200 registrants from 78 countries. Since then, the community has organized additional, similar training events, including COVID-19 data analysis workshops, plant transcriptomics, machine learning, and single-cell analysis. To support this modality of training, we have also recently created the GTN video library (https://gallantries.github.io/video-library/).

### New communities

Increasingly, special interest groups (SIGs) are forming within the Galaxy community, centered around a physical location or a scientific domain: https://galaxyproject.org/community/. Recent examples include formation of Galaxy India and Galaxy Arabic speaking communities, with goals of creating local expertise as well as translating Galaxy Training Materials to local languages (https://training.galaxyproject.org/training-material/news/2021/05/20/spanish_project_begins.html). SIGs have also formed around specific domains that focus on adding documentation, training, wrapping tools, and features to accommodate their use cases. Recent examples include biology-focused groups focused on cheminformatics (18), single-cell RNA-Seq (19) and public health (20) as well as climate science as an all-new domain (https://climate.usegalaxy.eu/).

### Transparent project governance

The continuous growth and diversity of needs in the communities meant a more scalable governance model was needed. The project formed a Galaxy Steering Committee to collect the high-level views, interests, and needs of the communities. Working Groups were formed as assemblies of contributors that focus on realizing set agendas. The Executive Board is charged with overseeing project procedures and is responsible for the creation of the community roadmap involving all stakeholders. This explicit and open governance structure allows anyone to join a working group and start shaping the future of the project.

## FUTURE PLANS

The growing Galaxy project community will continue to push the boundaries of data science at the level of compute infrastructure, novel models of user interaction, and large science projects. Here we highlight a few new initiatives:

### Data-local computing

Galaxy manages the datasets that users analyze by storing a local copy of each dataset. Storing copies is becoming untenable as biomedical datasets grow and are distributed across local and cloud repositories. We are now working on support for data-local computing where Galaxy will only store a universally unique identifier (UUID) for a dataset and the data for a given analysis step will be fetched as required. This will reduce the resources needed for analyses and enable operating on public and private repositories.

### Novel user interfaces

The Galaxy ‘history’ panel, which displays the progression of a user’s analysis in a linear succession of datasets, has remained relatively unchanged since 2006. This linear view becomes limiting as the number of datasets and analysis complexity continues to grow. We are actively creating a novel history interface that is designed to scale and gracefully handle 10 000 datasets while giving a better sense and insight into the analysis flow through graph and ‘minimap’ style modes of interaction.

### Scientific partnerships

Historically, genome assembly was not broadly accessible, requiring technical expertise, high-quality data, and powerful computational resources. The Vertebrate Genome Project (VGP) aims to change this using the latest sequencing technologies and developing new assembly tools, with the goal of assembling reference genomes for all 71,657 known vertebrate species (21). Galaxy has partnered with the VGP to develop genome assembly workflows (https://bit.ly/3KXmgWY) that are available on free, accessible public infrastructure (6). In addition to reducing costs and increasing throughput for VGP, these workflows are universally available for any genomics researcher, ushering in a new era of reference genomes and pan-genomes. Similar partnerships are being pursued in cancer genomics, proteomics, chemoinformatics, climate change (https://galaxyproject.org/use/).

## Notes

https://galaxyproject.org/.

## APPENDIX

**Corresponding author:** Enis Afgan^18^ (enis.afgan@jhu.edu)

**Co-corresponding authors**: Anton Nekrutenko^25^ (anton@nekrut.org), Bjórn A Grüning^35^ (bjoern.gruening@gmail.com), Daniel Blankenberg^6^ (dan.blankenberg@gmail.com), Jeremy Goecks^23^ (goecksj@ohsu.edu), Michael C Schatz^18^ (mschatz@cs.jhu.edu)

### Contributors (alphabetical)

Alexander E Ostrovsky^18^, Alexandru Mahmoud^18^, Andrew J Lonie^36^, Anna Syme^36^, Anne Fouilloux^33^, Anthony Bretaudeau^15^, Anton Nekrutenko^25^, Anup Kumar^35^, Arthur C Eschenlauer^34^, Assunta D DeSanto^25^, Aysam Guerler^18^, Beatriz Serrano-Solano^35^, Bérénice Batut^35^, Björn A Grüning^35^, Bradley W Langhorst^22^, Bridget Carr^18^, Bryan A Raubenolt^6^, Cameron J Hyde^26^, Catherine J Bromhead^36^, Christopher B Barnett^29^, Coline Royaux^21^, Cristóbal Gallardo^35^, Daniel Blankenberg^6^, Daniel J Fornika^2^, Dannon Baker^18^, Dave Bouvier^25^, Dave Clements^1^, David A de Lima Morais^39^, David Lopez Tabernero^35^, Delphine Lariviere^25^, Engy Nasr^35^, Enis Afgan^18^, Federico Zambelli^37^, Florian Heyl^35^, Fotis Psomopoulos^7^, Frederik Coppens^40^, Gareth R Price^38^, Gianmauro Cuccuru^35^, Gildas Le Corguillé^28^, Greg Von Kuster^25^, Gulsum Gudukbay Akbulut^25^, Helena Rasche^11^, Hans-Rudolf Hotz^13^, Ignacio Eguinoa^40^, Igor Makunin^26^, Isuru J Ranawaka^17^, James P Taylor^18^, Jayadev Joshi^6^, Jennifer Hillman-Jackson^25^, Jeremy Goecks^23^, John M Chilton^25^, Kaivan Kamali^25^, Keith Suderman^18^, Krzysztof Poterlowicz^4^, Le Bras Yvan^21^, Lucille Lopez-Delisle^12^, Luke Sargent^23^, Madeline E Bassetti^26^, Marco Antonio Tangaro^14^, Marius van den Beek^25^, Martin Čech^16^, Matthias Bernt^30^, Matthias Fahrner^35^, Mehmet Tekman^35^, Melanie C Föll^35^, Michael C Schatz^18^, Michael R Crusoe^41^, Miguel Roncoroni^40^, Natalie Kucher^18^, Nate Coraor^25^, Nicholas Stoler^25^, Nick Rhodes^38^, Nicola Soranzo^9^, Niko Pinter^35^, Nuwan A Goonasekera^36^, Pablo A Moreno^10^, Pavankumar Videm^35^, Petera Melanie^15^, Pietro Mandreoli^37^, Pratik D Jagtap^34^, Qiang Gu^23^, Ralf J.M. Weber^3^, Ross Lazarus^27^, Ruben H.P. Vorderman^20^, Saskia Hiltemann^11^, Sergey Golitsynskiy^18^, Shilpa Garg^19^, Simon A Bray^35^, Simon L Gladman^36^, Simone Leo^8^, Subina P Mehta^34^, Timothy J Griffin^34^, Vahid Jalili^5^, Vandenbrouck Yves^31^, Victor Wen^18^, Vijay K Nagampalli^6^, Wendi A Bacon^24^, Willem de Koning^11^, Wolfgang Maier^35^, Peter J Briggs^42^

### Affiliations

^1^ Anaconda Inc., Austin, TX, USA

^2^ BC Centre for Disease Control Public Health Laboratory, Vancouver, British Columbia, Canada

^3^ University of Birmingham, Birmingham, West Midlands, UK

^4^ University of Bradford, Bradford, West Yorkshire, UK

^5^ Broad Institute, Cambridge, MA, USA

^6^ Cleveland Clinic, Cleveland, OH, USA

^7^ Centre for Research and Technology Hellas, Thessaloniki, Greece

^8^ Center for Advanced Studies, Research, and Development in Sardinia, Pula, CA, Italy

^9^ Earlham Institute, Norwich, Norfolk, UK

^10^ EMBL European Bioinformatics Institute, Hinxton, Cambridgeshire, UK

^11^ Erasmus Medical Center, Rotterdam, Netherlands

^12^ Ecole Polytechnique Fédérale de Lausanne, Lausanne, Switzerland

^13^ Friedrich Miescher Institute for Biomedical Research, Basel, Switzerland

^14^ Institute of Biomembranes and Bioenergetics, Bari, Italy

^15^ National Research Institute for Agriculture, Food and Environment, Rennes, France

^16^ IOCB Prague, Prague, Chech Republic

^17^ Indiana University, Bloomington, IN, USA

^18^ Johns Hopkins University, Baltimore, MD, USA

^19^ University of Copenhagen, Copenhagen, Copenhagen, Denmark

^20^ Leiden University Medical Center, Leiden, Netherlands

^21^ National Museum of Natural History, Concarneau, France

^22^ New England Biolabs, Ipswich, MA, USA

^23^ Oregon Health & Science University, Portland, OR, USA

^24^ The Open University, Milton Keynes, Buckinghamshire, UK

^25^ Pennsylvania State University, State College, PA, USA

^26^ Queensland Cyber Infrastructure Foundation, Brisbane, Queensland, Australia

^27^ Galaxy Emeritus, Sydney, NSW, Australia

^28^ Station Biologique de Roscoff, Roscoff, France

^29^ University of Cape Town, Cape Town, Western Cape, South Africa

^30^ Helmholtz Centre for Environmental Research, Leipzig , Germany

^31^ Université Grenoble Alpes, Grenoble, France

^32^ Ghent University, Ghent, Belgium

^33^ University of Oslo, Oslo, Norway

^34^ University of Minnesota, Minneapolis, MN, USA

^35^ University of Freiburg, Freiburg, Baden-Württemberg, Germany

^36^ University of Melbourne, Melbourne, Victoria, Australia

^37^ University of Milan, Milan, Italy

^38^ University of Queensland, Saint Lucia, Queensland, Australia

^39^ Université de Sherbrooke, Sherbrooke, Quebec, Canada

^40^ VIB Center for Plant Systems Biology, Ghent, Belgium

^41^ Vrije Universiteit Amsterdam (VU Amsterdam), Berlin, Germany

^42^ University of Manchester, Manchester, North West England, UK
