# Correction to ‘The Galaxy platform for accessible, reproducible and collaborative biomedical analyses: 2022 update’

**DOI:** 10.1093/nar/gkac610

**Published:** 2022-07-29

*Nucleic Acids Research*, gkac247, https://doi.org/10.1093/nar/gkac247

In the published paper, p.6, References, reference 13 was erroneous and should read:

‘Goble, C., Soiland-Reyes, S., Bacall, F., Owen, S., Williams, A., Eguinoa, I., Droesbeke, B., Leo, S., Pireddu, L., Rodríguez-Navas, L., *et al.* (2021) Implementing FAIR digital objects in the EOSC-Life workflow collaboratory. Extended abstract, Zenodo doi: https://doi.org/10.5281/zenodo.4605654, 12 March 2021, preprint: not peer reviewed.’

instead of

‘Silva, R. F.d., Pottier, L., Coleman, T., Deelman, E. and Casanova, H. (2020) WorkflowHub: Community Framework for Enabling Scientific Workflow Research and Development. In: *2020 IEEE/ACM Workflows in Support of Large-Scale Science (WORKS)*. pp. 49-56.’

These errors have been corrected in the article.

